# Molecular characterisation of a novel citrus-infecting emaravirus, citrus emaravirus 1

**DOI:** 10.1007/s00705-025-06424-0

**Published:** 2025-10-08

**Authors:** R. Bester, C. Gill, J.H.J Breytenbach, C. Steyn, H.J. Maree, G. Cook

**Affiliations:** 1 Citrus Research International, PO Box 2201, Matieland, 7602 South Africa; 2 Department of Genetics, Stellenbosch University, Private Bag X1, Matieland, 7602 South Africa; 3 Citrus Research International, P.O. Box 28, Nelspruit, 1200 South Africa

**Keywords:** *Fimoviridae*, *Elliovirales*, Chlorotic ring symptoms, Negative-sense RNA virus, Citrus virome

## Abstract

The application of high-throughput sequencing (HTS) has accelerated the discovery of novel viruses and the genome sequencing of poorly characterised viruses in fruit crops, revealing a greater complexity of plant viromes than previously understood. Here, we report the identification and genomic characterisation of a novel emaravirus, tentatively named "citrus emaravirus 1" (CiEV1), from *Citrus limon* leaves collected in the North West Province of South Africa. HTS analysis identified contigs corresponding to the four conserved emaravirus genomic segments (RNA1-RNA4), each encoding characteristic proteins: RNA-dependent RNA polymerase, glycoprotein, nucleocapsid, and movement protein. Comparison of terminal sequences and phylogenetic analysis suggest placing CiEV1 within clade D of the genus *Emaravirus* (family *Fimoviridae*). To date, no association with symptoms has been identified. This is the first report of an emaravirus detected in citrus, expanding the known host range of the genus and contributing to the growing evidence of viral diversity in citrus. Further investigation is required to determine the epidemiological significance of CiEV1 in citrus orchards.

**Supplementary Information:**

The online version contains supplementary material available at 10.1007/s00705-025-06424-0.

Citrus is one of the most economically important fruit crops globally, cultivated across subtropical and tropical regions for fresh consumption and juice production [1]. Genomes of citrus viruses with economic impact, such as citrus tristeza virus [2, 3] and citrus yellow vein clearing virus [4, 5] have been characterised using high-throughput sequencing (HTS) technologies. HTS has additionally enabled the discovery of citrus viruses as aetiological agents of disorders that were unresolved for decades, such as concave gum [6] and impiertratura [7]. More broadly, HTS has accelerated the discovery of previously undetected viruses, particularly those with atypical genome structures or low titres. One such virus group is the genus *Emaravirus* (family *Fimoviridae*) [8, 9], a lineage of negative-sense RNA viruses primarily known to infect woody perennials and ornamentals, often associated with unique symptoms including leaf deformation, ringspots, and chlorosis. To date, only a limited number of emaravirus species have been reported in fruit trees, and none have been associated with citrus infections. The latest report of the International Committee on Taxonomy of Viruses (ICTV) (August 2024) includes 33 species in the genus *Emaravirus* (https://ictv.global/taxonomy). Emaraviruses have segmented genomes, usually with between four and ten distinct RNA segments [8, 10, 11]. The first four core segments include RNA1 to RNA4, which code for an RNA-dependent RNA polymerase (RdRp), a glycoprotein precursor (GP), a nucleocapsid protein (NP), and a movement protein (MP), respectively. A characteristic feature of these viruses is the presence of complementary sequences at the 5′ and 3′ ends of each RNA segment that are potentially capable of forming a panhandle structure [9, 11, 12]. Some fimoviruses can also be transmitted naturally by eriophyid mites [12–15]. Here, we report the identification and genomic characterization of a putative new emaravirus, tentatively named "citrus emaravirus 1" (CiEV1), isolated from citrus.

Concentric ring blotch (CRB) of citrus occurs sporadically in some parts of southern Africa, including South Africa, and is associated with the presence of citrus grey mites (*Calacarus citrifolii* Keiffer) in *Citrus limon* (lemon), *Citrus reticulata* (mandarin) hybrids, and *Citrus sinensis* (sweet orange) [16], but no aetiology has been ascribed to CRB. It is not a disease of commercial importance; however, identification of a pathogen would enable development of diagnostic assays to distinguish CRB from other diseases that might display similar symptomatology. In November 2022, citrus leaves displaying irregular, chlorotic ring-shaped patterns typical for CRB (Fig. 1) were collected from orchards in the North West Province of South Africa. Similar symptoms were observed on lemon, mandarin, and navel orange leaves. No fruit symptoms were observed or reported from the sampled orchards. Total RNA was extracted using a CTAB extraction protocol [17, 18] from symptomatic and asymptomatic leaf material from representative trees of each citrus type. Total RNA from seven symptomatic leaf samples (five from lemon and two from mandarin) were analysed using Illumina high-throughput sequencing (HTS) as described previously [19]. Analysis of the HTS data revealed contigs with sequence similarity to members of the genus *Emaravirus* in all five of the lemon samples. Only four different emaravirus-like contigs with sequence similarity to the four core segments of emaraviruses (RNA1-RNA4) were identified in the samples. The closest match in the NCBI GenBank database was camellia chlorotic ringspot virus (MT040095.1; 68% identity and 44% query coverage for RNA1). *De novo*-assembled contigs were analysed using BLASTn and BLASTx against a local GenBank nt database, a custom virus database, and an emaravirus-specific database, with reads also mapped to emaravirus references, but no additional emaravirus-related sequences were detected beyond the four contigs with similarity to RNA1-4 of camellia chlorotic ringspot virus. The sample with the highest number of reads associated with the emaravirus-like contigs was selected for genome sequence assembly and sequence validation through the design of primers to produce overlapping amplicons (Supplementary Information S1).

**Fig. 1:**
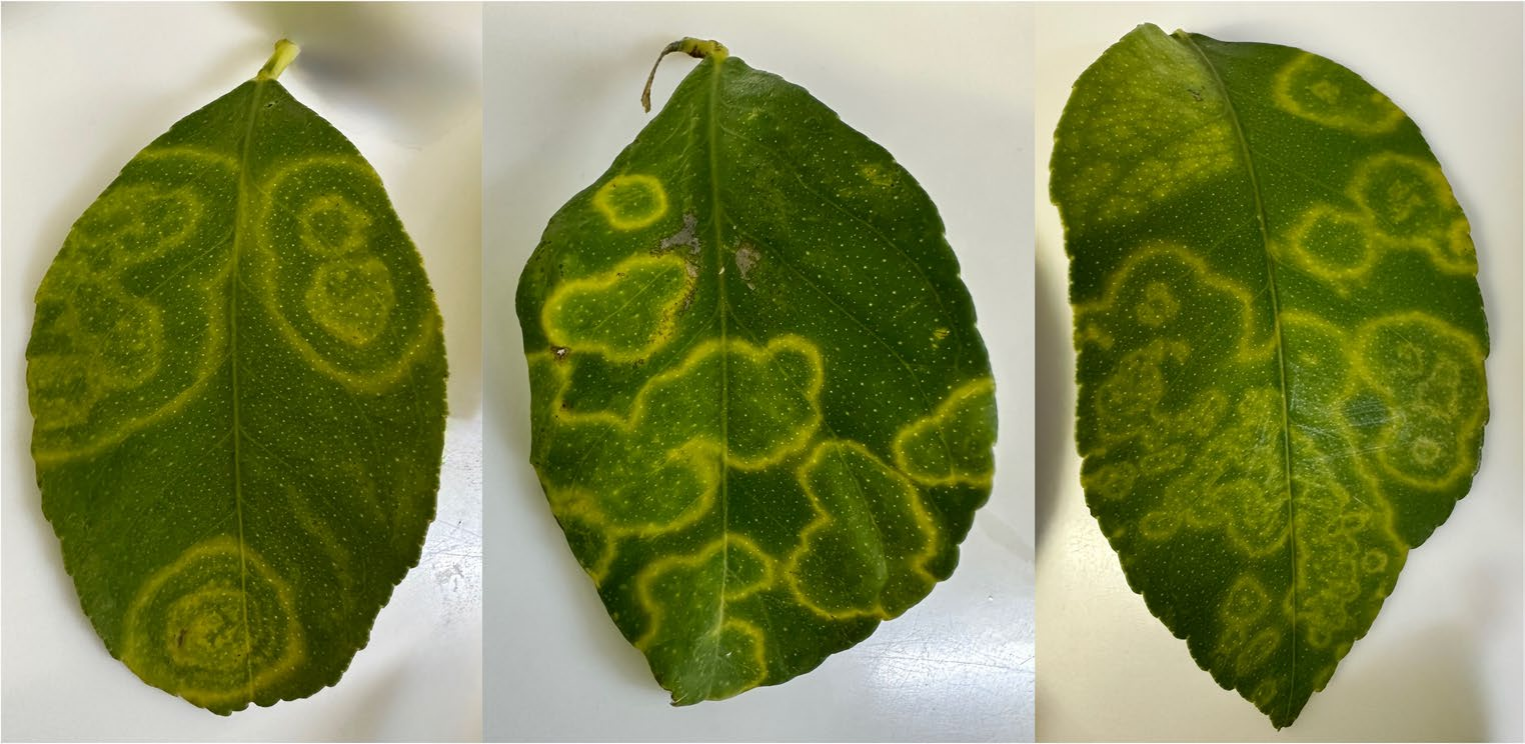
Lemon (*Citrus limon*) leaves displaying irregular chlorotic ring-shaped patterns

Complementary DNA (cDNA) was synthesized from total RNA from sample ER1S, using random hexamer primers (Promega) and Maxima Reverse Transcriptase (Thermo Fisher Scientific), following the manufacturer’s instructions. Complementary DNA was amplified using genome-specific forward and reverse primers (IDT) (primer sets 1–10, Supplementary Information S1) and KAPA Taq DNA Polymerase (Kapa Biosystems, Roche) with KAPA Taq Buffer A, following the manufacturer’s instructions. DNA from the amplicons of interest were sent to the Central Analytical Facility (CAF) of Stellenbosch University for bidirectional Sanger sequencing using the primers used for amplification. To amplify the 5’ and 3’ ends of the RNA segments, an initial attempt was made to amplify the full-length sequences by RT-PCR using the primer pair 5H/3C [20]. This was, however, unsuccessful, providing the first indication that this citrus emaravirus might not belong to clades A, B, or C [8]. Subsequently, the 5’ and 3’ end sequences were obtained by polyadenylation of total RNA using *E. coli* poly(A) polymerase (New England Biolabs) according to manufacturer’s instructions, followed by reverse transcription to generate cDNA using an oligo d(T) primer (primer 27, Supplementary Information S1). The cDNA was subjected to thermal cycling as mentioned above, using the oligo d(T) primer and genome-specific reverse and forward primers for the amplification of the 5’ and 3’ end, respectively (primers 11–25, Supplementary Information S1). DNA from the amplicon of interest was cloned into pGEM-T Easy Vector (Promega), and plasmid DNA was sent to CAF at Stellenbosch University for Sanger sequencing using T7 and SP6 primers. The final sequences of the genome segments of the CiEV-1 segments of isolate ER1S were assembled from the overlapping amplicons using CLC Main Workbench 7 (QIAGEN) and were deposited in the GenBank database under the accession numbers PV823284-PV823287. ORFfinder (https://www.ncbi.nlm.nih.gov/orffinder/) was used to predict open reading frames (ORFs).

A modified version of primer PDA213 [21] (primer 26, Supplementary Information S1) was used for cDNA synthesis using Maxima Reverse Transcriptase and PCR using PrimeSTAR GXL DNA Polymerase (Takara) to amplify additional sequences in the sample. Only amplicons corresponding to the lengths of RNA2-4 were obtained. Primer 26 (Supplementary Information S1) was also used in combination with primers 11–25 (Supplementary Information S1) to confirm the 5’ and 3’ ends of the four genome segments.

The CiEV1 genome was found to consist of at least four negative-sense RNAs, each containing a single ORF. RNA1 is 7175 nt in length (PV823284) and contains a large ORF (nt positions 7118 to 129) with similarity to the RdRps of other emaraviruses. All seven conserved motifs (Pre-A, F, and A-E) that have been identified in other members of the family *Fimoviridae* [22, 23] are present in RNA1 of CiEV1. RNA2 is 1595 nt in length (PV823285), with an ORF that encodes a glycoprotein (nt positions 1514 to 114). RNA3 is 1291 nt in length (PV823286) and contains one ORF from nt positions 1143 to 265 that encodes a protein with similarity to the putative nucleocapsid proteins of other emaraviruses. RNA4 is 1408 nt in length (PV823287) and has an ORF from nt positions 1318 to 278 that encodes a putative movement protein. The 5′ (AGTAGTT-) and 3′ termini (-TCATCAA) of CiEV1 RNAs are conserved and are similar to those found in members of phylogenetic clade D of emaraviruses [8].

To determine the phylogenetic position of CiEV1 within the order *Elliovirales*, a multiple sequence alignment of the amino acid sequences of the RdRp was performed using MAFFT version 7 [24], and a maximum-likelihood (ML) tree was constructed using IQ-Tree [25] with automatic model selection and 1000 ultra-fast bootstrap replicates. The tree was visualized using FigTree v1.4.4. Phylogenetic analysis based on RdRp amino acid sequences (Fig. 2) showed CiEV1 clustering in the previously described clade D of the genus *Emaravirus*, and it is most closely related to members of the species *Emaravirus verbanni* and *Emaravirus camelliae.* Based on the sequence diversity of the genome segments and the differences in the conservation in their 5’ and 3’ ends [8], it can be proposed that clade D should be subdivided (Fig. 2).

**Fig. 2:**
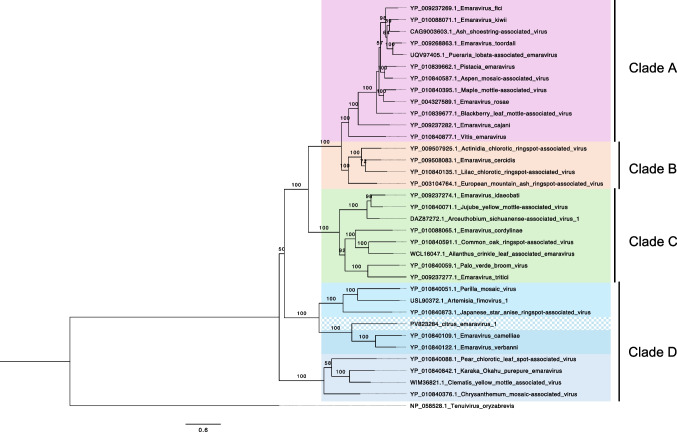
A maximum-likelihood (ML) tree inferred from a multiple sequence alignment of the RNA-dependent RNA polymerase (RdRp) amino acid sequence of citrus emaravirus 1 (CiEV1) and RdRp sequences of members of the genus *Emaravirus* obtained from the GenBank database*.* The substitution model was selected automatically (LG+F+I+G4), and 1000 ultra-fast bootstrap replicates were performed. The final tree was visualised using FigTree, and a representative of the species *Tenuivirus oryzabrevis* was included as an outgroup. Branch lengths represent the number of substitutions per site. Bootstrap values of 50 and higher are displayed. The position of CiEV1 is highlighted with a patterned block, and the diversity observed in clade D is indicated with different shades of blue.

According to the species demarcation criterion for the genus *Emaravirus*, two out of the four amino acid sequences of the RdRp, GP, NP, and MP should differ by >20% (https://ictv.global/report/chapter/fimoviridae/fimoviridae/emaravirus. Because CiEV1 has the highest sequence identity of 42%, 24%, 29%, and 58% to the RdRp, GP, NP, and MP, respectively, of a representative of the species *Emaravirus camelliae* (YP_010840109.1, YP_010840111.1, YP_010840113.1, YP_010840110.1), CiEV1 should be considered a member of a new species in the genus *Emaravirus*, or even a new genus of the family *Fimoviridae*, as proposed previously [8].

To investigate the association between the irregular chlorotic ring-shaped patterns observed on leaves and the presence of CiEV1, eight symptomatic and eight asymptomatic lemon leaf samples from eight different trees were screened for the presence of CiEV1 using RT-PCR (primer set 1, Supplementary Information S1). Leaves from six mandarin trees and one navel orange tree were also sampled to obtain a symptomatic sample and an asymptomatic sample from each tree, and these were screened using primer set 1. Symptomatic and asymptomatic areas on the same leaf were also sampled. Only the eight symptomatic samples and the samples containing the symptomatic lesions from the lemon trees tested positive for CiEV1 while the symptomatic mandarin orange hybrid and navel orange samples tested negative, including the asymptomatic parts of the leaves with lesions. The sampled leaves had concentric ring blotches, but no associated fruit symptoms were observed. Symptomatic leaves from the same lemon orchards and mandarin hybrid orchard as well as an additional orchard were sampled in 2023 and tested for CiEV1 by RT-PCR. The results confirmed that CiEV1 was only detected in lemon lesions. The detection of CiEV1 only in foliar ring blotches of lemon and not in similar lesions sampled from the other citrus types suggests that the presence of the virus might be an incidental association, contained as a local lesion-infection, and that the chlorotic ring patterns observed are not necessarily associated with the presence of CiEV1. Whether CiEV1 can induce chlorotic ring patterns in lemon still needs to be assessed.

In summary, a novel virus, tentatively named "citrus emaravirus 1" (CiEV1), was identified in lemon trees, using HTS. Analysis of the validated genome sequences showed typical features of members of the genus *Emaravirus* (family *Fimoviridae*), but according to the demarcation criterion for the genus *Emaravirus* it was concluded that CiEV1 is a member of a distinct species. This finding expands the known host range of emaraviruses and adds to the growing body of evidence that citrus viromes are more diverse than previously recognised.

## Supplementary Information

ESM1 (DOCX 27 kb)

## Data Availability

The datasets generated during and/or analysed during the current study are available from the corresponding author on reasonable request.
