# Supplementary material for: Molecular characterisation of a novel citrus-infecting emaravirus, citrus emaravirus 1
Source: Arch Virol. 2025 Oct 8;170(11):219. doi: 10.1007/s00705-025-06424-0 (PMC12507997; doi:10.1007/s00705-025-06424-0)
Supplement: Supplementary file 1 — (DOCX 27 kb) [file 705_2025_6424_MOESM1_ESM.docx]

**Complete genome sequence of a novel citrus-infecting emaravirus, citrus emaravirus 1 (CiEV1)**

R Bester^1,2*^, C. Gill^1^, J.H.J Breytenbach^3^, C. Steyn^3^, H.J. Maree^1,2^ and G. Cook^3^

^1^Citrus Research International, PO Box 2201, Matieland, 7602, South Africa

^2^Department of Genetics, Stellenbosch University, Private Bag X1, Matieland, 7602, South Africa

^3^Citrus Research International, P.O. Box 28, Nelspruit, 1200, South Africa

*Corresponding author: [rachelle@sun.ac.za](mailto:rachelle@sun.ac.za)

| Primer pair/number | Primer name | Primer sequence (5'-3') |
| --- | --- | --- |
| 1 | C1638_RNA1_F | CCCAGTGTATGAGGCTAAATC |
|  | C1638_RNA1_R | GAGAAGAGGCAATAACAACACC |
| 2 | C192_RNA2_F | GGCAATGTTTCCTATCTCAAGC |
|  | C192_RNA2_R | AGGCAAACAAAGCGTGGATG |
| 3 | C783_RNA3_F | TTCCAATACTCCTATTCCATGA |
|  | C783_RNA3_R2 | AAGCACCTAAAAGTTCAACAGY |
| 4 | C2150_RNA4_F2 | GGGTTCCATTAGGGTCTGAYT |
|  | C2150_RNA4_R | TGCCTTGGTTCTGGTATCTTTC |
| 5 | Emara_RNA1_944F | TGTACCTCACTTTAGTTTGTCTTA |
|  | Emara_RNA1_2258R | AGGATCATGGATTCAAACAAAGC |
| 6 | Emara_RNA1_1958F | AATTTAATTCATACCTTGTGCCAT |
|  | Emara_RNA1_3071R | ATAATGATGGGTGATGAATTAACTT |
| 7 | Emara_RNA1_2925F | CAAGTATGGGCAAAGATGTGAA |
|  | Emara_RNA1_4013R | ACCAAAGTGATGGAAGAAGTTTA |
| 8 | Emara_RNA1_3767F | GTGTTGTTATTGCCTCTTCTCTT |
|  | Emara_RNA1_4748R | AGTGGATTAAAAGTTTCTTCTGGT |
| 9 | Emara_RNA1_4471F | CTAATTTCACTTGCCCTATCTAA |
|  | Emara_RNA1_5574R | TGAACTTATGATAGGTCCAAATGA |
| 10 | Emara_RNA1_5407F | ATTGTGCTCATGGTTATTATGGAT |
|  | Emara_RNA1_6399R | TGATGGATTTACGATGACAGATGT |
| 11 | Emara_RNA1_6136F | TCATCATCTAATAAGGTTGTTGGC |
| 12 | Emara_RNA1_211R | AGACTGCTTACAGCTTCTTGGGGGATGA |
| 13 | Emara_RNA1_1191R | CATGATACTGAATTACTGGTCCAA |
| 14 | Emara_RNA1_134F | CATCATCCCCCAAGAAGCTGT |
| 15 | Emara_RNA1_1552R | TCGATAGATCCAAGATGAACACTAA |
| 16 | Emara_RNA2_1390F | ATCATTATTGGGTCTATTCCTATCAT |
| 17 | Emara_RNA2_480R | TGCTTGAGATAGGAAACATTGCC |
| 18 | Emara_RNA3_125R | AGGTGAAATAAGATAATAAGAGTAGT |
| 19 | Emara_RNA3_349F | ACCAGGTCTTTGTCGAAAATGG |
| 20 | Emara_RNA3_442F | TCAGGCTTTCAACTTTGGTCA |
| 21 | Emara_RNA3_1111R | AAGCTATCTGAAAGTTCAAAAGAGTT |
| 22 | Emara_RNA4_1093F | AACAGTCCAAAGCAAAGCCAAC |
| 23 | Emara_RNA4_1394F | TGGACTACATTTGACTATTAGGCATT |
| 24 | Emara_RNA4_ 118F | TTTATCACAGCCTTTTATTCTTGT |
| 25 | Emara_RNA4_ 1405R | AACTACCTTGTAACTGATAAAGAT |
| 26 | PDAP213_S2Fs | GGCGACCCGCTCCGGTACCCTAGTAGTT |
| 27 | GVA-dT(17) | TACGATGGCTGCAGTTTTTTTTTTTTTTTTT |
